# Elementary Practical Biology, Vegetable

**Published:** 1887-09

**Authors:** 


*Elementary Practical Biology, Vegetable.* By THOMAS W. SHORE, M.D., B.Sc. Lond., Lecturer on Comparative Anatomy at St. Bartholomew's Hospital. London: J. & A. CHURCHILL. 1887.

For those who are about seriously to work in the laboratory at the acquirement of the knowledge of the structure of the various types from the vegetable kingdom, required by the London University in its examination in Elementary Biology, Dr. Shore has provided an excellent manual. It is thoroughly practical, thoroughly scientific, and thoroughly trustworthy, and will prove the best-arranged existing guide to the student who is devoting his attention to the study of Practical Vegetable Biology—that is to say, to the structural anatomy of plants, both coarse and microscopical; for though the term biology carries with it a physiological suggestion, of physiology proper there is almost nothing. In order that the book may not be used for the purposes of "cram," Dr. Shore has avoided the insertion of all illustrations or even diagrams: this exalted moral position will be wholesome in its influence upon the students, but had Dr. Shore pandered a little to popularity in this direction, the book would have appealed more warmly to those botanists who are not students preparing for examination. May, however, the author's virtue meet with the reward it deserves!

